# MicroRNA 744-3p promotes MMP-9-mediated metastasis by simultaneously suppressing PDCD4 and PTEN in laryngeal squamous cell carcinoma

**DOI:** 10.18632/oncotarget.11280

**Published:** 2016-08-13

**Authors:** John Zeng-Hong Li, Wei Gao, Wen-Bin Lei, Jing Zhao, Jimmy Yu-Wai Chan, William Ignace Wei, Wei-Kuen Ho, Thian-Sze Wong

**Affiliations:** ^1^ Department of Surgery, The University of Hong Kong, Queen Mary Hospital, Hong Kong; ^2^ Department of Otolaryngology, The First People's Hospital of Foshan, Foshan, People's Republic of China; ^3^ Department of Otolaryngology, The First Affiliated Hospital of Sun Yet-Sen University, Guangdong, People's Republic of China

**Keywords:** miR-744-3p, metastasis, PDCD4, PTEN, laryngeal squamous cell carcinoma

## Abstract

MicroRNA controls cancer invasion by governing the expression of gene regulating migration and invasion. Here, we reported a novel regulatory pathway controlled by miR-744-3p, which enhanced expression of matrix metallopeptidase 9 (MMP-9) in laryngeal squamous cell carcinoma (LSCC). We profiled the differential micoRNA expression pattern in LSCC cell lines and normal epithelial cultures derived from the head and neck mucosa using microRNA microarray. MiR-7-1-3p, miR-196a/b and miR-744-3p were expressed differentially in the LSCC cell lines. Subsequent validation using real-time PCR revealed that high miR-744-3p level was positively correlated with regional lymph node metastasis of LSCC. Real-time cellular kinetic analysis showed that suppressing miR-744-3p could inhibit migration and invasion of LSCC cell lines and reduce the number of lung metastatic nodules in nude mice modules. *In silico* analysis revealed that miR-744-3p targeted 2 distinct signaling cascades which eventually upregulated MMP-9 expression in LSCC. First, miR-744-3p could suppress programmed cell death 4 (PDCD4), a direct suppressor of NF-κB (p65). PDCD4 could also prevent AKT activation and suppress MMP-9 expression. Further, suppressing miR-744-3p expression could restore phosphatase and tensin homolog (PTEN) expression. PTEN could inhibit AKT activation and inhibit MMP-9 expression in LSCC cells. The results revealed that suppressing miR-744-3p was effective to inhibit LSCC metastasis by inactivating AKT/mTOR and NF-κB (p65) signaling cascade. Targeting miR-744-3p could be a valuable therapeutic intervention to suppress the aggressiveness of LSCC.

## INTRODUCTION

Cancer of the larynx originating from the head and neck region is the second most common upper-aerodigestive cancer [1, 2]. Histologically, laryngeal squamous cell carcinoma (LSCC) is the major form. Despite the advances of cancer treatment in the past few decades, the prognosis of LSCC patients remains poor without significant improvement [3]. Recent data reveals that the morbidity and mortality associates with LSCC are increasing in recent year [3, 4]. Local invasion and metastatic spread of cancer cells is one of the main causes of death in LSCC patients [5]. Laryngeal cancer patients present with cervical lymph node involvement have poorer disease free interval and overall survival [6]. Given that the key cellular and molecular mechanisms resulting in LSCC migration and invasion are less understood, investigation on the underlying mechanism will be clinically beneficial for developing molecular targeting therapy.

MicroRNAs are highly conserved non-coding RNAs (~18–25 nucleotides) involved in gene regulation at post-transcription level. The single-stranded mature microRNA can bind to the target mRNA at the 3′ untranslated region (3′UTR) forming a stable duplex by Watson-Crick complementarities at a partial complementary manner. By recruiting and incorporating with the RNA-induced silencing complex (RISC), translation of the target mRNA transcript is hindered and/or the mRNA is subjected to degradation [7, 8]. MicroRNA is recognized as a master gene regulator as individual microRNA could target a plethora of mRNA transcript at multiple sites the same time [9]. This multiple target feature makes microRNA an efficient effector in target-based cancer therapy which offers unique advantages as compared with the conventional treatment modalities [10].

In order to identify the key functional microRNA in LSCC, we performed microRNA profiling in LSCC cell lines using microRNA microarray and validated the results in LSCC tissues. We found that deregulation of miR-744- 3p was a common event in LSCC. At present, however, its functions in LSCC remains to be defined. *In silico* analysis showed that miR-744-3p could directly target the mRNA transcript of both programmed cell death 4 (PDCD4) and phosphatase and tensin homolog (PTEN), both of which had been reported to be correlated with LSCC metastasis [11–13]. Reduced PDCD4 was usually found in aggressive head and neck cancers [14]. PDCD4 knock-out mice showed high systematic dissemination rate implying the functional implication in the metastatic process [15, 16]; PTEN, on the other hand, was a well-known anti-neoplastic factor [17], which antagonized the action of PI3K by converting PIP3 to PIP2 via dephosphorylation [18]. Both PTEN and PDCD4 were identified as the upstream suppressors of matrix metallopeptidase 9 (MMP-9) which facilitated cancer cell migration through degrading the collagenous substrates in the surrounding extracellular matrix [19]. Our results revealed a novel pathway employed by LSCC in promoting LSCC migration and metastasis by overexpressing miR-744-3p.

## RESULTS

### MicroRNA expression patterns in the LSCC and normal epithelial cell lines

**Figure 1:**
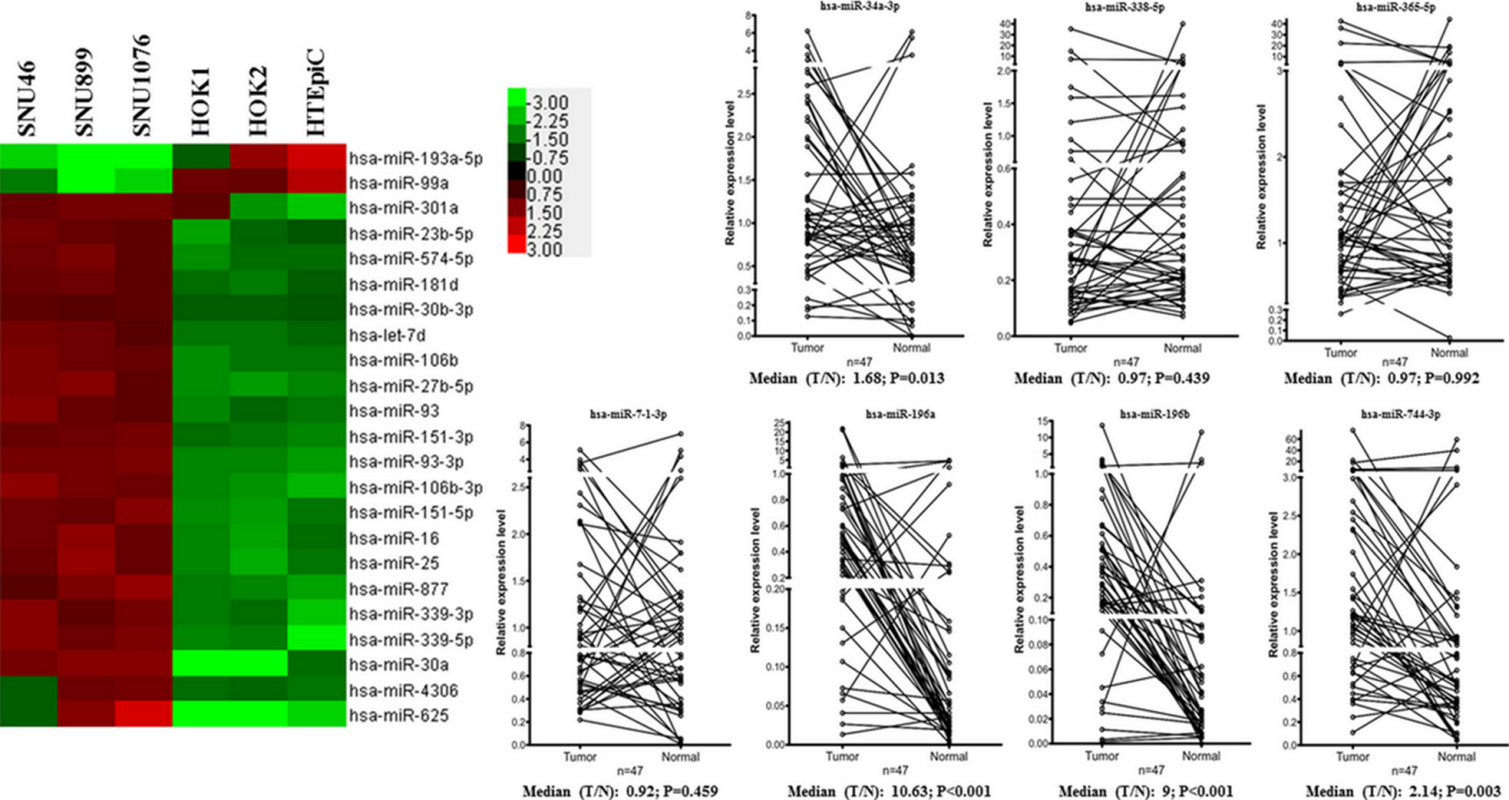
Deregulated microRNAs in LSCC As shown in the heat map, 21 microRNAs overexpressed and 2 microRNAs were downregulated in LSCC cell lines respectively. The line charts showed the expression levels of the 8 differentially expressed microRNAs in LSCC tissues. MiR-196a, miR-196b and miR-744-3p were significantly overexpressed in LSCC compared with their paired normal counterparts (*P* < 0.05).

Table 1 showed the deregulated microRNA expression profile in LSCC cell lines. MiR-7-1-3p, miR- 196a, miR-196b and miR-744-3p were detected in LSCC but not normal epithelial cultures. In comparison, let-7a-3p, miR-34a-3p, miR-338-5p and miR-365a- 5p could only be detected in normal epithelial culture. Twenty-three microRNAs showed significant difference in expression level between the LSCC and normal cell lines (1.5-fold, *P* < 0.05) (Figure 1). The microarray data are publicly available at GEO (Accession No. GSE73171).

**Table 1:** Expression of deregulated microRNAs in LSCC cell lines and normal epithelial culture revealed by microarray profiling

ProbeSet Name	Detectable in tumor cell lines	Detectable in normal epithelial culture	Mean Ratio (T/N)	*P* value (*T* test)
hsa-let-7a-3p	No	Yes	-----	-----
hsa-miR-338-5p	No	Yes	-----	-----
hsa-miR-34a-3p	No	Yes	-----	-----
hsa-miR-365a-5p	No	Yes	-----	-----
hsa-miR-193a-5p	Yes	Yes	0.1724	0.03559
hsa-miR-99a	Yes	Yes	0.2983	0.02728
hsa-miR-4306	Yes	Yes	1.5112	0.04447
hsa-miR-181d	Yes	Yes	1.5870	0.00419
hsa-miR-23b-5p	Yes	Yes	1.6129	0.03223
hsa-let-7d	Yes	Yes	1.6737	0.01306
hsa-miR-574-5p	Yes	Yes	1.7924	0.01606
hsa-miR-93	Yes	Yes	1.7939	0.01544
hsa-miR-151-3p	Yes	Yes	1.8870	0.00048
hsa-miR-301a	Yes	Yes	1.889	0.03815
hsa-miR-106b	Yes	Yes	1.9559	0.00079
hsa-miR-16	Yes	Yes	2.1452	0.01156
hsa-miR-151-5p	Yes	Yes	2.2301	0.00450
hsa-miR-339-3p	Yes	Yes	2.2357	0.00998
hsa-miR-25	Yes	Yes	2.2572	0.01428
hsa-miR-93-3p	Yes	Yes	2.3123	0.00004
hsa-miR-877	Yes	Yes	2.3139	0.01860
hsa-miR-27b-5p	Yes	Yes	2.3741	0.00401
hsa-miR-106b-3p	Yes	Yes	2.6147	0.00230
hsa-miR-339-5p	Yes	Yes	2.6273	0.00340
hsa-miR-30a	Yes	Yes	3.2300	0.00383
hsa-miR-625	Yes	Yes	5.1155	0.04386
hsa-miR-196a	Yes	No	-----	-----
hsa-miR-196b	Yes	No	-----	-----
hsa-miR-7-1-3p	Yes	No	-----	-----
hsa-miR-744-3p	Yes	No	-----	-----

Twenty-three microRNAs showed significant difference in expression level between the LSCC and normal cell lines (1.5– fold, *P* < 0.05). MiR-7-1-3p, miR-196a, miR-196b and miR-744-3p were detected in LSCC but not normal epithelial cultures. In comparison, let-7a-3p, miR-34a-3p, miR-338-5p and miR-365a-5p could only be detected in normal epithelial culture.

### MiR-744-3p was overexpressed in LSCC tissues

Next, we validated the microarray results by analyzing the aberrant expressed microRNA level (miR-7-1-3p, miR- 196a, miR-196b, miR-744-3p, let-7a-3p, miR-34a- 3p, miR-338-5p and miR-365a-5p) in a cohort of 47 LSCC tissues using QPCR and compared with the paired normal tissues (Figure 1).

Three microRNA (miR-196a, miR-196b and miR- 744-3p) were significantly upregulated in the LSCC tissue (*P* < 0.05). Let-7a-3p was not detected in all the LSCC tissues and the paired normal epithelia. MiR- 7-1-3p, miR-338-5p and miR-365a-5p were detected in the LSCC tissues and the paired normal epithelia. However, there were no significant difference in the expression level between cancer and the normal tissues (*P* > 0.05). In the microarray results, miR-34a-3p expression was found in normal epithelial cell lines and was undetectable in the LSCC cell lines. In the validation set using laryngeal tissues, however, miR-34a-3p was significantly upregulated in tumor (*P* = 0.013). Thus, we shortlisted miR-196a, miR-196b and miR-744- 3p as candidate microRNA and explored their clinical significance by evaluating the statistical association with the clinicopathological parameters of LSCC patients.

All the LSCC cases were grouped into high expression and low expression group using median expression level of each microRNA in LSCC as cut- off points. As shown in Table 2, expression levels of miR- 196a and miR-196b were not statistically associated with the age, smoking habit, drinking habit, T-stage, regional lymph node status, or clinical stage of our LSCC patients. In contrast, high miR-744-3p expression was significantly associated with the cervical lymph node metastasis in LSCC (*P* = 0.007). Thus, we proposed that miR-744-3p overexpression could promote migration and invasion of the LSCC cells.

**Table 2:** Clinical findings and expression of miR-196a, miR-196b and miR-744-3p in LSCC

		miR-196a			miR-196b			miR-744-3p		
	Total	High level	Low level	*P* value	High level	Low level	*P* value	High level	Low level	*P* value
**Total**	47	24	23		24	23		24	23	
**Age**										
**≥ 60**	24	13	11	0.664	14	10	0.308	14	10	0.308
**< 60**	23	11	12		10	13		10	13	
**Smoking**										
**Yes**	36	20	16	0.265	21	15	0.071	18	18	0.792
**No**	11	4	7		3	8		6	5	
**Alcohol consumption**										
**Yes**	17	10	7	0.423	11	6	0.159	10	7	0.423
**No**	30	14	16		13	17		14	16	
**T stage**										
**T1/T2**	17	9	8	0.846	10	7	0.423	8	9	0.679
**T3/T4**	30	15	15		14	16		16	14	
**Lymph node metastasis**										
**Yes**	15	9	6	0.401	7	8	0.68	12	3	**0.007**
**No**	32	15	17		17	15		12	20	
**Clinical stage**										
**I + II**	12	5	7	0.45	7	5	0.559	4	8	0.154
**III + IV**	35	19	16		17	18		20	15	

Using the median of individual microRNA expression level as cut-off point, the LSCC cases were allocated into high level and low level groups regarding the corresponding microRNA expression level. Pearson Chi square test was adopted to examine the correlation between the clinical feature and each microRNA expression level. The high miR-744-3p expression level could significantly increase the risk of lymph node metastasis in LSCC.

### Suppressing miR-744-3p reduced the metastatic ability of LSCC

**Figure 2:**
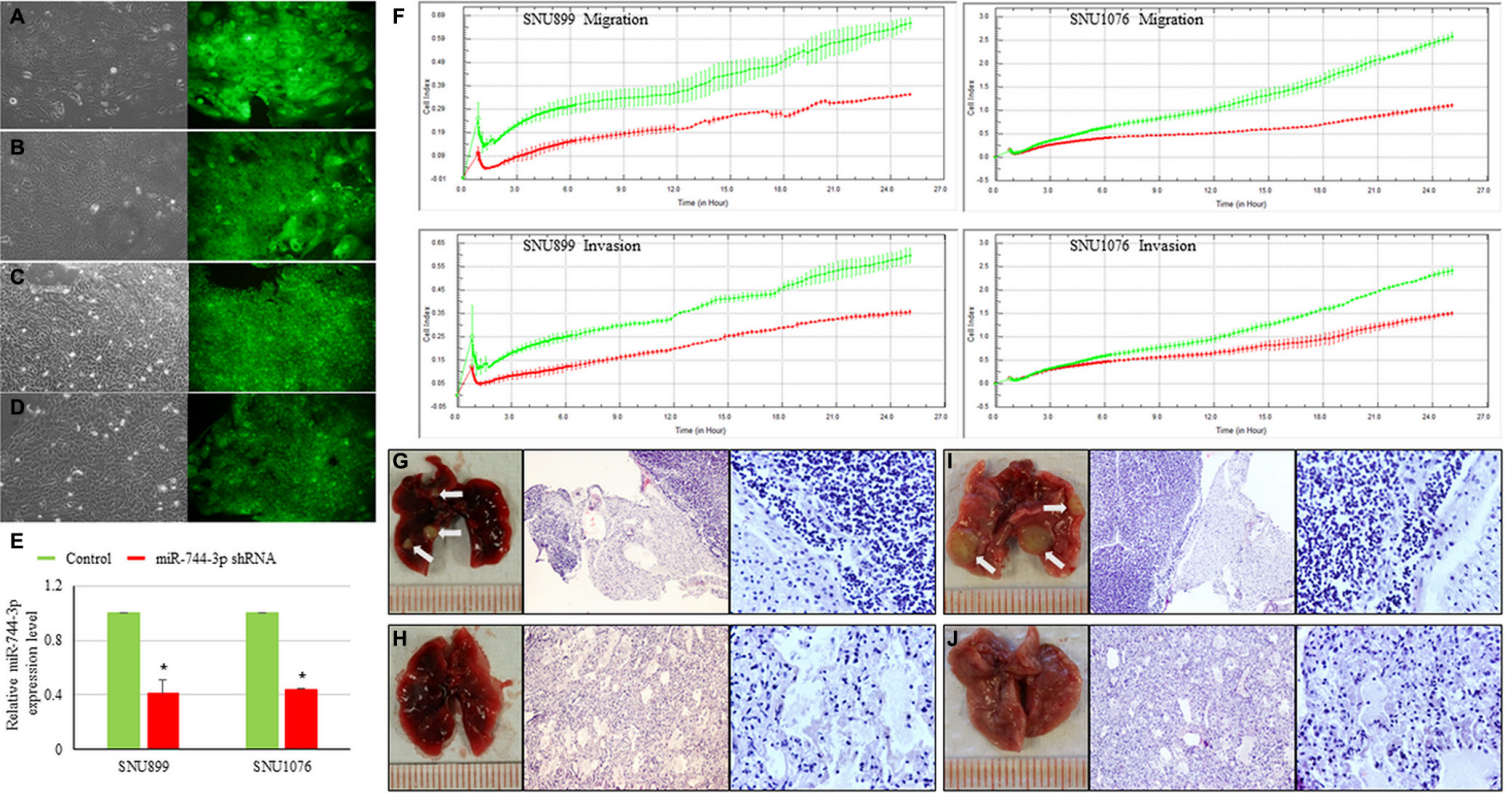
Influence of miR-744-3p suppression on LSCC metastasis (**A–D**) Figures of LSCC cell lines with mock control vector or expressing miR-744-3p shRNA. The left part of each figure is the bright field image of each clone, while the right part is the green fluorescent image of cells. (A) SNU899 with mock control vector; (B) SNU899 stably expressing miR-744-3p shRNA; (C) SNU1076 with mock control vector; (D) SNU1076 stably expressing miR-744-3p shRNA. (**E**) Relative expression levels of miR-744-3p. Compared with the mock control, the stable LSCC clone compromising miR-744-3p shRNA exhibited significant reduction of miR-744-3p expression level. (**F**) Real-time measurement of LSCC cell migration and invasion. The reduction of miR-744-3p could significantly inhibit the LSCC cell migration and invasion. (**G– J**) Metastatic foci in lung of representative nude mice injected with miR-744-3p suppressing LSCC cell lines and mock control (H&E staining). G: SNU899 with mock control vector; (H) SNU899 stably expressing miR-744-3p shRNA; (I) SNU1076 with mock control vector; (J) SNU1076 stably expressing miR-744-3p shRNA. Compared with the mock control groups, less metastatic foci were observed in the mice injected with miR-744-3p silencing LSCC cells. White arrow: metastatic foci. *P < 0.05, **P < 0.01.

To explore the functional impact of miR-744-3p overexpression on LSCC migration and invasion, we monitored the dynamic changes of migration and invasion capacities of LSCC cell lines (SNU899 and SNU1076) expressing the miR-744-3p shRNA. Significant reduction in migration and invasion ability was observed in both SNU899 and SNU1076 (Figure 2F). To confirm the *in vitro* data, the miR-744-3p suppressed LSCC cell line was injected into nude mice and the formation of lung metastatic foci were evaluated on day 60 after injection. Mice injected with miR-744-3p suppressed LSCC cells group had lower number of metastatic cancer nodules in comparison with the control group (Figure 2G–2J).

### *In silico* analysis showed that miR-744-3p targeted PDCD4 and PTEN simultaneously

To evaluate the functional target of miR-744- 3p, the target transcript was predicted using miRWalk. In total, 2004 genes were simultaneously predicted by 2 or more databases. Of which, 13 genes had been reported to be downregulated in LSCC tissues [20–28]. Next, we used the mirSVR score to narrow down the gene list. Six transcripts (PDCD4, PTEN, SCN2B, IGFBP5, GPR64, BOC) had mirSVR score < −0.01 suggesting that they could potentially form thermodynamically stable complexes with the mature miR-744-3p (Table 3). Among the 6 genes, only PDCD4 and PTEN were reported to consistently suppress head and neck cancer cell metastasis in previous studies. PTEN restoration could significantly suppress migration and invasion in LSCC and oral carcinoma. On the other hand, low PDCD4 expression was significantly related with lymph node metastasis in head and neck malignancy, and PDCD4 upregulation could inhibit invasiveness of oral cancer and nasopharyngeal carcinoma cells [29–34].

**Table 3:** Genes consistently suggested as potential targets of miR-744-3p and downregulated transcripts in LSCC

Gene	Sequence	Dataset	mirSVR score
AKT2	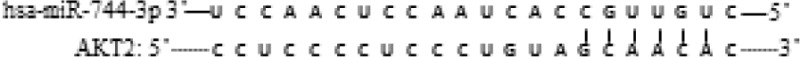	DIANAmT, miRanda	−0.0005
EMP1	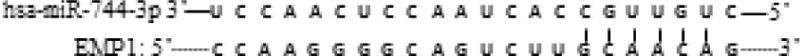	miRanda, miRWalk	−0.0057
ADH1B	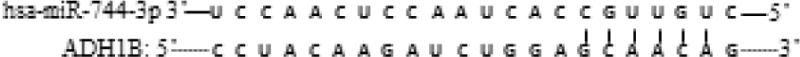	miRanda, miRWalk	−0.0063
ADH1C	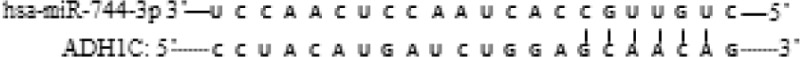	miRanda, miRWalk	−0.0162
MS4A1	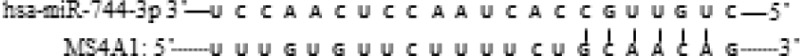	miRanda, miRWalk	−0.0187
CLDN10	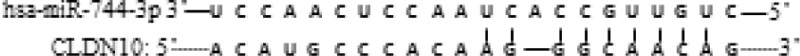	miRanda, miRWalk	−0.0491
ATOH8	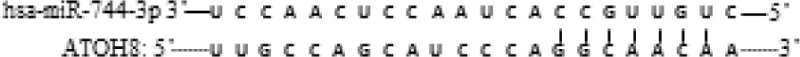	miRanda, miRWalk	−0.0993
PDCD4	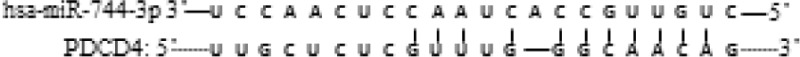	miRanda, miRWalk	−0.2355
PTEN	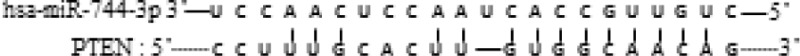	miRanda, miRWalk	−0.3082
SCN2B	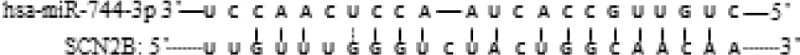	miRanda, miRWalk	−0.3613
IGFBP5	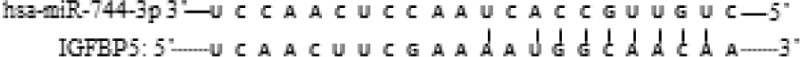	miRanda, miRWalk	−0.4013
GPR64	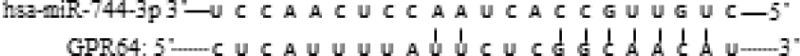	miRanda, miRWalk	−0.4579
BOC	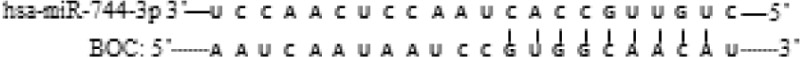	miRanda, miRWalk	−1.1002

In total 13 potential target genes of miR-744-3p were retrieved, which were also downregulated in LSCC according to literature review.

### Reduced PDCD4 and PTEN expression were correlated with the regional nodal status of LSCC patients

**Figure 3:**
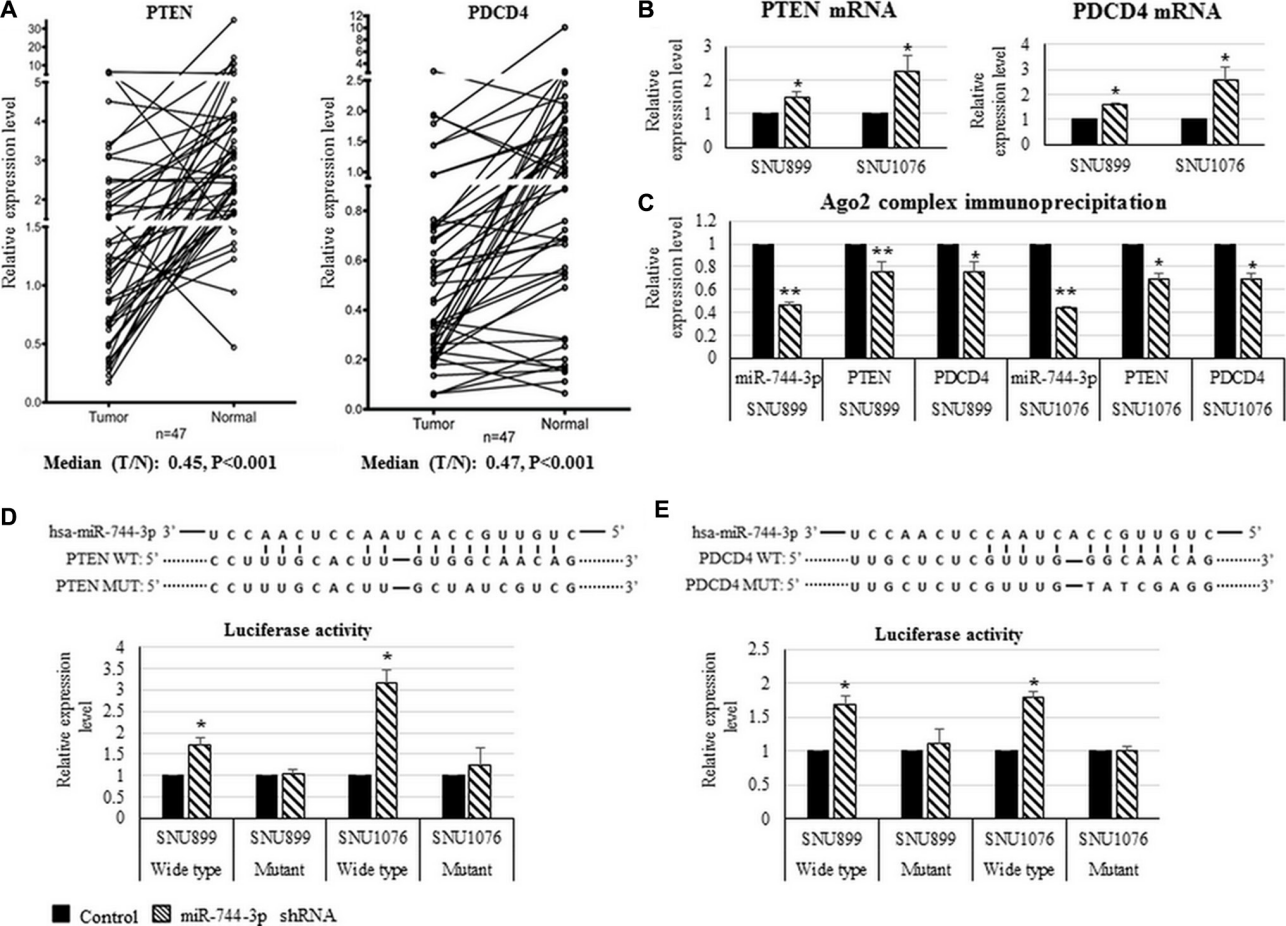
Expression of PTEN and PDCD4 transcripts and their interaction with miR-744-3p in LSCC (**A**) Relative expression levels of PTEN mRNA and PDCD4 mRNA in LSCC. Compared with the normal epithelial counterparts, the tumor exhibited significantly lower PTEN mRNA and PDCD4 mRNA expression levels. (**B**) Relative expression levels of PTEN mRNA and PDCD4 mRNA in LSCC cell lines. By miR-744-3p suppression, expression levels of PTEN and PDCD4 were significantly increased in both of SNU899 and SNU1076. (**C**) Ago 2 protein immunoprecipitation. Compared with mock control group, the suppression of miR-744- 3p significantly reduced miR-744-3p, PTEN and PDCD4 binding to the Ago2 protein. (**D, E**) Relative luminenscent signal intensity in luciferase reporter assay. The suppression of miR-744-3p significantly enhanced luciferase expression from the reporter plasmid containing the wild- type PTEN 3′-UTR sequence or containing the wild- type PDCD4 3′-UTR sequence. Similar enhancement of luminenscent signal intensity due to miR-744-3p suppression was not observed in the groups with mutant PTEN 3′-UTR sequence or mutant PDCD4 3′-UTR sequence. *P < 0.05, **P < 0.01.

In the LSCC tissues, PDCD4 and PTEN expression were significantly reduced (Figure 3A). Low PDCD4 (*P* = 0.037) and PTEN (*P* < 0.001) were significantly associated with the regional lymph node metastasis of LSCC patients. Reduced PTEN expression was also found to be significantly associated with the advanced clinical stage (*P* = 0.036). Furthermore, MiR-744-3p was negatively correlated with PTEN (R = −0.319, *P* = 0.029) and PDCD4 (R = −0.574, *P* < 0.001) in LSCC tissues (Table 4).

**Table 4:** Correlation between PTEN, PDCD4 mRNA expression and clinical characteristics of LSCC patients as well as miR-744-3p expression

	PDCD4			PTEN			MMP-9		
	High level	Low level	*P* value	High level	Low level	*P* value	High level	Low level	*P* value
Total	23	24		23	24		24	23	
Age									
≥ 60	9	15	0.109	8	16	**0.029**	14	10	0.387
< 60	14	9		15	8		10	13	
Smoking									
Yes	20	16	0.101	18	18	0.792	17	19	0.494
No	3	8		5	6		7	4	
Alcohol consumption									
Yes	7	10	0.423	13	11	0.159	6	11	0.135
No	16	14		17	6		18	12	
T stage									
T1/T2	9	8	0.679	10	7	0.307	10	7	0.547
T3/T4	14	16		13	17		14	16	
Lymph node metastasis									
Yes	4	11	**0.037**	1	14	**< 0.001**	12	3	**0.011**
No	19	13		22	10		12	20	
Clinical stage									
I + II	8	4	0.154	9	3	**0.036**	5	7	0.517
III + IV	15	20		14	21		19	16	
Correlation with miR-744-3p (Pearson correlation)									
High miR-744-3p expression level	5	19	***R* = −0.574**	**8**	**16**	***R* = −0.319**	**14**	**10**	*R* = 0.149
Low miR-744-3p expression level	18	5	***P* < 0.001**	**15**	**8**	***P* = 0.029**	**10**	**13**	*P* = 0.387

Using the median expression level of PTEN, PDCD4 and MMP-9 mRNA in LSCC tissues as cut-off value, the patients were divided into high expression level group and low expression level group. Low PTEN expression in LSCC was significantly correlated with lymph node metastasis and late clinical stage. Low PDCD4 expression in LSCC was significantly correlated with lymph node metastasis. High MMP-9 expression in LSCC was significantly correlated with lymph node metastasis. Furthermore, the expression level of PTEN and PDCD4 were negatively correlated with miR-744-3p expression level.

### MiR-744-3p targeted PDCD4 and PTEN transcript in LSCC

As shown in Figure 3B, suppressing miR-744-3p in the LSCC cell lines (SNU899 and SNU1076) resulted in significant increase in PTEN and PDCD4 transcript expression (*P* < 0.05). Target gene prediction analysis revealed that PDCD4 and PTEN 3′-UTR harbored the binding sites for miR-744-3p seed sequence. In the Ago2-IP/IgG-IP, PDCD4 and PTEN mRNA levels were reduced when the endogenous expression of miR-744-3p were suppressed by shRNA (Figure 3C). The association between miR-744-3p and PDCD4 or PTEN was further validated using luciferase activity reporter containing PDCD4 or PTEN 3′-UTR. As shown in Figure 3D and 3E, there was a significant increase in the luciferase activity when miR-744-3p was suppressed endogenously. In the mutant construct of which miR-744-3p binding sequence on the PDCD4 or PTEN 3′-UTR were disrupted, the luciferase activity remained stable with no significant changes.

### Suppression of miR-744-3p reduced AKT and NF-κB (p65) activation through PTEN and PDCD4 enhancement in LSCC

**Figure 4:**
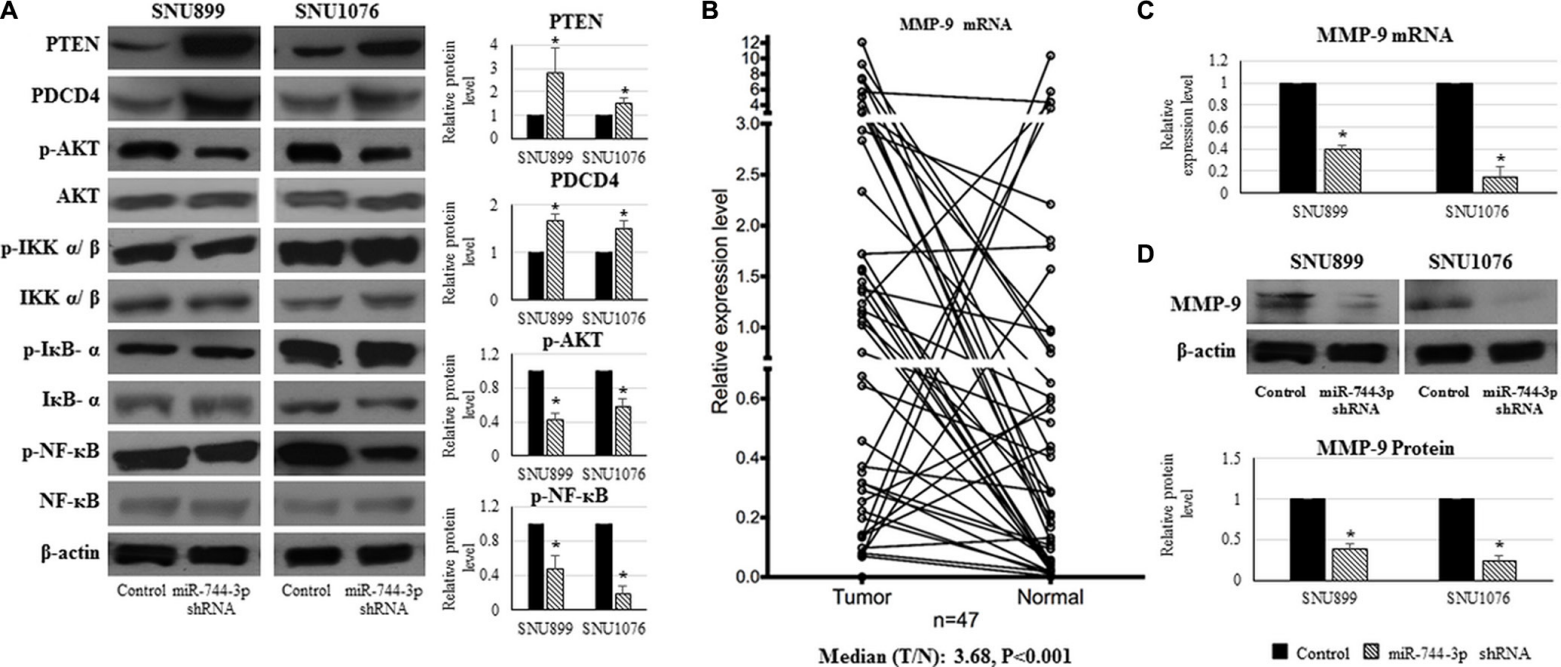
Expression alteration of PTEN, PDCD4, phosphorylated AKT, phosphorylated NF-κB and MMP-9 caused by miR-744-3p suppression in LSCC (**A**) Western blot analysis on protein expression in LSCC cell lines. By miR-744-3p suppression, PTEN and PDCD4 protein levels were significantly increased, while phosphorylated AKT and phosphorylated NF-κB protein levels were significantly reduced. (**B**) Relative expression levels of MMP-9 mRNA in LSCC. Compared with the normal epithelial counterparts, the tumor exhibited significantly higher MMP-9 mRNA expression level. (**C**) Relative expression levels of MMP-9 mRNA in LSCC cell lines. By miR-744-3p suppression, MMP-9 mRNA expression level was significantly reduced in both of SNU899 and SNU1076. (**D**) MMP-9 protein expression levels in LSCC cell lines. By miR-744-3p suppression, MMP-9 protein level was significantly reduced. *P < 0.05, **P < 0.01.

In SNU899 and SNU1076 with miR-744-3p suppression, the levels of PTEN and PDCD4 proteins were significantly increased, while phosphorylated AKT and phosphorylated NF-κB (p65) protein levels were significantly reduced. The phosphorylated IKKα/β and IκB-α level remained unchanged in the miR-744-3p suppressed LSCC cell lines (Figure 4A).

### MMP-9 expression was reduced in miR-744-3p silencing LSCC

MMP-9 expression was significantly upregulated in the LSCC tissues (*P* < 0.001, Figure 4B). High MMP-9 expression was significantly associated with the regional lymph node metastasis of LSCC patients (*P* = 0.011, Table 4).

As activated AKT and NF-κB (p65) could promote MMP-9 upregulation in head and neck cancers, we hypothesized that the miR-744-3p modulated MMP- 9 expression in LSCC. As shown in Figure 4C and 4D, MMP- 9 mRNA and protein level were remarkably reduced in miR-744-3p suppressed SNU899 and SNU1076 (*P* < 0.05).

### PDCD4 restoration reduced the metastatic ability of LSCC

**Figure 5:**
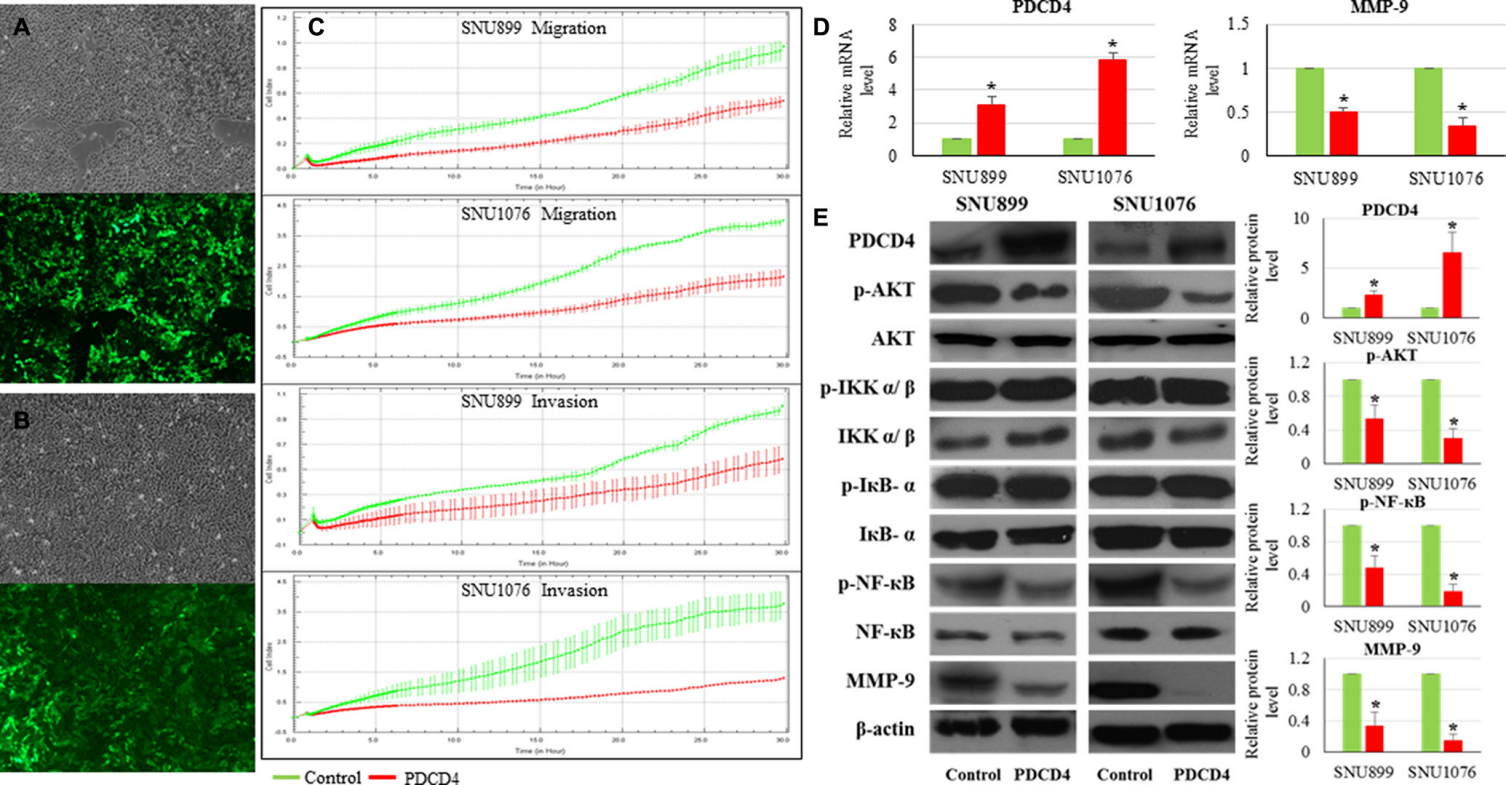
Influence of PDCD4 restoration on LSCC (**A**) SNU899 stably expressing PDCD4; (**B**) SNU1076 stably expressing PDCD4. The upper part of figure is the bright field image of each clone, while the lower part is the green fluorescent image of cells. (**C**) Real-time measurement of LSCC cell migration and invasion. PDCD4 restoration could inhibit LSCC cell migration and invasion. (**D**) Relative expression levels of PDCD4 and MMP-9 mRNA in LSCC cell lines. Compared with the mock control, the stable LSCC clone with PDCD4 restoration exhibited significant enhancement of PDCD4 and reduction of MMP-9 mRNA expression levels. (**E**) Protein expression levels of PDCD4, phosphorylated AKT, phosphorylated NF-κβ and MMP-9 in LSCC cell lines. Compared with the mock control, PDCD4 protein level was significantly increased, while phosphorylated AKT, phosphorylated NF-κβ and MMP-9 protein levels were significantly reduced.

As a famous tumor suppressor gene, PDCD4 was described as inhibitor of cancer cell migration and metastasis in different cancer forms including LSCC. Low PDCD4 expression was observed to be significantly related with poor differentiation and lymph node metastasis in LSCC patients [12, 13]. However, the mechanisms underlying its suppression remained to be determined. We continuously monitored the dynamic changes of migration and invasion capacities of LSCC cell lines with PDCD4 restoration. LSCC cell lines (SNU899 and SNU1076) expressing PDCD4 (Figure 5A and 5B) showed significant reduction in the migration and invasion ability in comparison with the mock controls (Figure 5C).

### PDCD4 restoration reduced AKT and NF-κB p65 activation as well as MMP-9 expression in LSCC

In the PDCD4 overexpressing clones, phosphorylated AKT and phosphorylated NF-κB (p65) levels were significantly reduced, while PDCD4 protein level was significantly increased (Figure 5E). The phosphorylated IKKα/β and IκB-α level remained unchanged in the LSCC cell lines with high PDCD4 expression (Figure 5E). Moreover, MMP-9 mRNA and protein level were remarkably reduced in SNU899 and SNU1076 with PDCD4 restoration (Figure 5D and 5E).

### MiR-744-3p shRNA showed higher efficacy than everolimus in suppressing MMP-9 expression in LSCC

**Figure 6:**
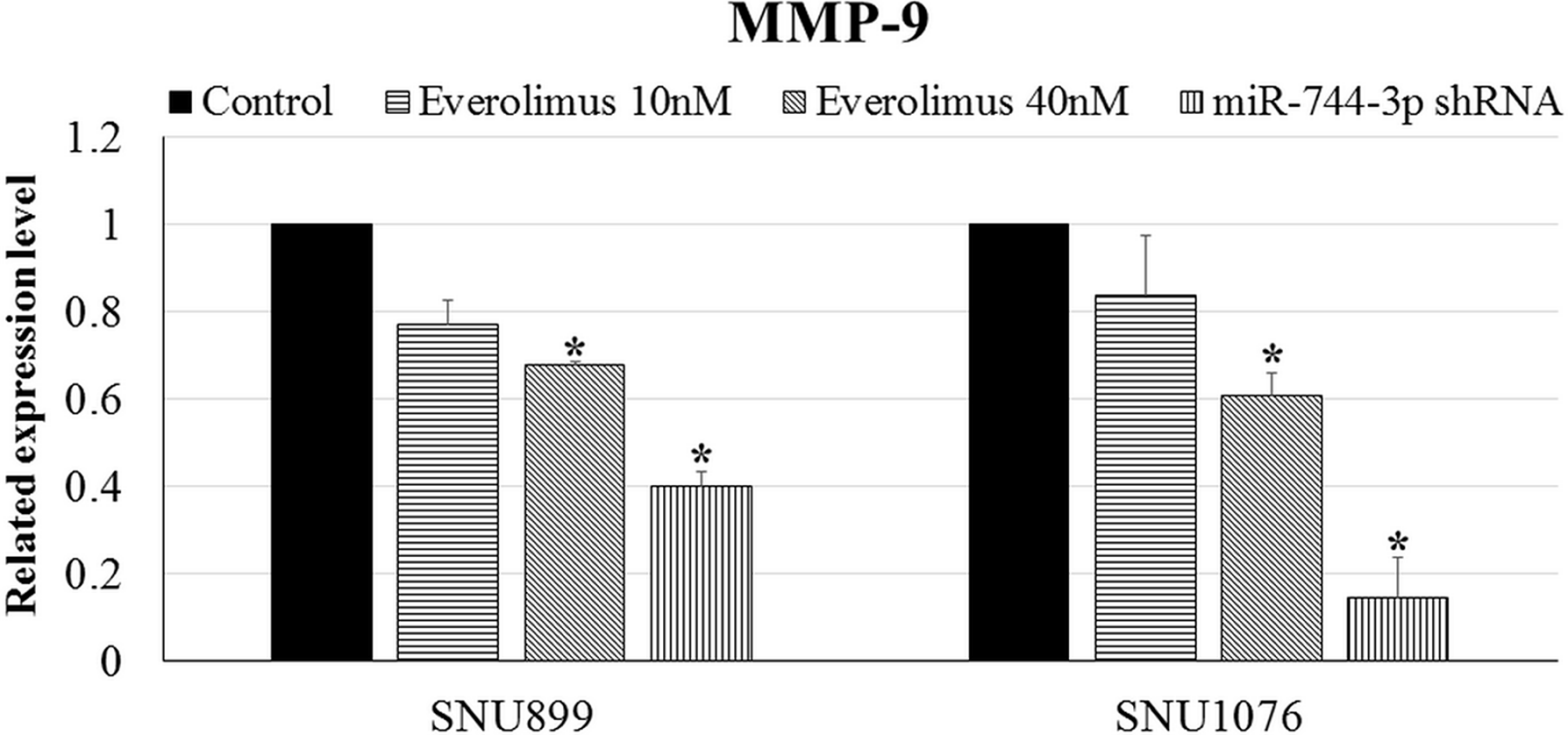
MMP-9 expression in LSCC cell lines Significant downregulation of MMP-9 mRNA expression level was observed in LSCC cell lines with miR-744-3p suppression. However, the reduction of MMP-9 mRNA was not significant in LSCC cell lines after administration of everolimus. *P < 0.05, **P < 0.01.

Everolimus is an mTOR inhibitor. Exposing LSCC to everolimus will lead to a reduction of the downstream gene regulated by mTOR signaling cascade. LSCC exposed to everolimus showed reduction in MMP-9 expression (Figure 6). MiR-744-3p shRNA, however, showed higher efficacy than everolimus in suppressing MMP-9 expression in LSCC (Figure 6). Compared with the mock control, MMP-9 mRNA level significantly decreased to 40% and 15% in SNU899 and SNU1076 when miR-744-3p shRNA were expressed in the LSCC cell lines (*P* < 0.05).

## DISCUSSION

As there was currently no data on the pathological functions of miR-744-3p, we first examined the tissue expression level and evaluated the statistical association with the clinicopathological characteristics of our LSCC patients. The association between high miR-744-3p expression and regional lymph node positive suggested that miR-744-3p could possibly play a part in controlling LSCC migration and invasion. To establish the causal relationship, we established the miR-744-3p suppressed LSCC cell lines and measured the changes in migration and invasion property of LSCC cells using real-time cellular kinetic assays and animal models. Our results confirmed that the expression level of miR-744-3p could have a functional impact on the migration and invasion propensity of LSCC cells.

MMP-9 expression is regulated by the upstream regulator PDCD4 and PTEN. MMP-9 could shape the development of epithelial–mesenchymal transition (EMT) phenotype in LSCC [35, 36]. Further, LSCC patients with low MMP-9 expression had a better 5-year survival rate [37]. In our LSCC samples, both PDCD4 and PTEN were significantly reduced. As miR-744-3p expression level in LSCC tissue was negatively correlated to the PDCD4 and PTEN transcript levels in the same tissue cohort, we proposed that miR-744-3p could possibly function as MMP-9 enhancer by inhibiting PDCD4 and PTEN expression in LSCC.

Our data showed that decreased PDCD4 was associated with the nodal status of LSCC patients indicating the role in LSCC migration and invasion. PDCD4 could suppress MMP-9 expression by inactivating AKT/mTOR signaling or reducing NF-κB (p65) activation [38, 39]. In the PDCD4 overexpressing LSCC cell lines, we noticed that the signal intensity of both pathways was reduced significantly. NF-κB (p65) was a critical oncogenic transcription factor. Activated NF-κB (p65) was involved in promoting cancer cell survival, inhibiting the apoptotic machinery, promoting angiogenesis and inducing cancer invasion [40]. PDCD4 could inactivate NF-κB(p65) and prevent NF-κB (p65)-mediated transcription activation of MMP-9 [41]. Hence, suppressing PDCD4 could promote cancer migration and invasion. PDCD4 could inactivate nuclear NF-κB (p65) without interfering the activation status of upstream IKKα/β and IκBα in brain tumor [41]. This was because PDCD4 could interact with NF-κB (p65) physically. Our data illustrated that the NF- κB (p65) inhibitory property of PDCD4 was effectively abolished by the high expression of miR-744-3p in LSCC.

The phosphatidylinositol 3 kinase (PI3K) / protein kinase B (PKB, also known as AKT) and mammalian target of rapamycin (mTOR) signaling cascade is the most commonly altered signaling cascade in head and neck carcinoma. PI3K (class I, II, and III) is a family of intracellular lipid and serine/threonine kinases. It functions by phosphorylating the downstream phosphatidylinositols and phosphoinositides at the 3′-hydroxyl group which triggers a cascade of signaling event. Upon receiving external stimulus, PI3K is recruited to the plasma membrane by the activated receptor and catalyzes the formation of phosphatidylinositol 3, 4, 5- bisphosphate (PIP3) from phosphatidylinositol 4, 5- bisphosphate (PIP2). PIP3 will activate the serine/threonine protein kinase Akt (Akt1, Akt2, and Akt3) and activate its activity by phosphorylating threonine 308 (by 3-phosphoinositide dependent kinase) or serine 473 (by rapamycin-insensitive mTOR complex/mTORC2) after binding AKT with its PH domain [18, 42, 43]. AKT and mTOR control key oncogenic signaling molecule including TP53, mitogen-activated protein kinase (MAPK)/ extracellular signal-regulated kinase, and eukaryotic initiation factor 4E (eIF4E) as well as NF-κB (p65) [44]. Phosphorylated AKT activates mTOR complex 1 (mTORC1), which later releases eIF4E by phosphorylation of eIF4E-binding protein (4EBP1). It has been shown that free eIF4E could activate MMP-9 expression [45]. In this signaling cascade, the tyrosine phosphatase PTEN functions as a central negative regulator which antagonize the action of PI3K by converting PIP3 to PIP2 via dephosphorylation [18] and hence reducing the quantity of activated AKT in the cells [46, 47]. It had been reported that reduced PTEN expression was associated with the deep invasion and lymph nodes metastasis of LSCC [11]. Low PTEN was observed in precancerous lesions suggesting that loss of PTEN was an early event in the development process of LSCC [27]. Targeting the PI3K/AKT/mTOR signaling pathway in head and neck cancer was suggested to be promising in improving the treatment efficacy and clinical outcome [48, 49]. Here, our results suggested that miR-744-3p could possibly control the mTOR signaling cascade by inhibiting PTEN expression in LSCC.

Everolimus is a selective inhibitor of mTOR approved by FDA for use in cancer treatment. As miR- 744- 3p could simultaneously target dual signaling channels responsible for MMP-9 activation, we hypothesized that suppressing miR-744-3p could have a therapeutic impact on inhibiting MMP-9 expression and should have a better efficacy than everolimus. In comparison with everolimus, MMP-9 expression was significantly reduced in miR-744-3p suppressed LSCC cell lines indicating that targeting miR-744-3p had a therapeutic impact in preventing MMP-9 upregulation in LSCC.

**Figure 7:**
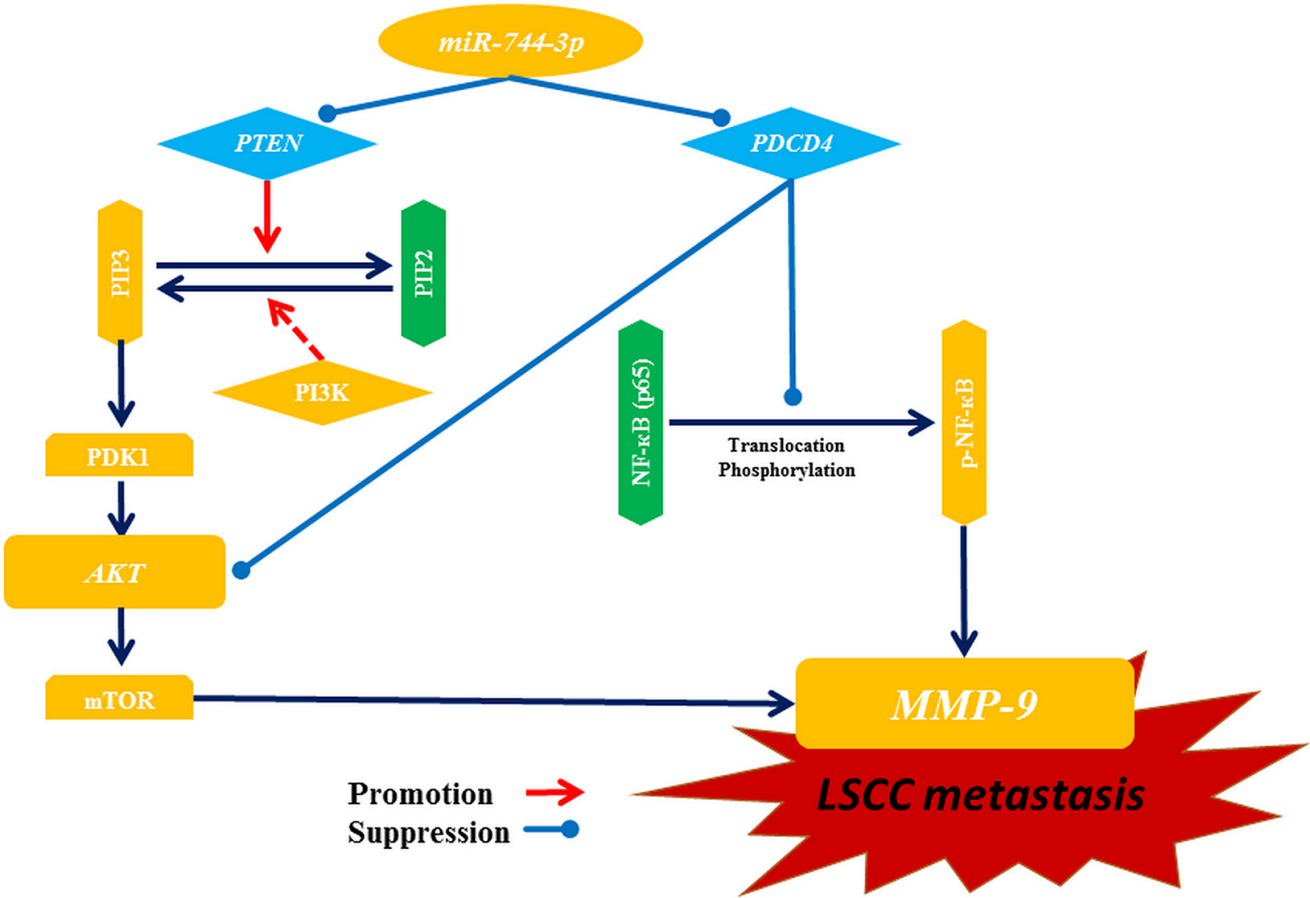
Illustration of potential pathways for miR-744-3p mediating metastasis in LSCC MiR-744-3p promotes LSCC metastasis by simultaneously inhibiting PTEN and PDCD4. Both of PTEN suppression and PDCD4 suppression can activate AKT and mTOR, and then further promote transcription activation of MMP-9, resulting in enhancement of LSCC metastasis. Moreover, loss of PDCD4 leads to NF-κB (p65) activation, which also promotes MMP-9 expression. Hence, suppression of miR-744-3p can powerfully prevent LSCC metastasis by inhibiting MMP-9 through inactivation of those 2 pathways above.

Collectively, our data suggested that miR-744-3p was an oncogenic microRNA in LSCC. On one hand, miR-744-3p suppressed the mTOR upstream regulatory element PTEN in the PI3K/AKT/mTOR regulatory axis, which controlled MMP-9 transcription. On the other hand, miR-744-3p activated another MMP-9 regulatory axis by provoking the signaling cascade controlled by NF-κB p65 via PDCD4 suppression (Figure 7). High miR-744-3p was crucial to activate MMP-9 expression in LSCC.

## MATERIALS AND METHODS

### Cell cultures and drug

LSCC cell lines (SNU46, SNU899, and SNU1076) were obtained from Korean Cell Line Bank [50]. Normal epithelial cultures derived from oral cavity epithelia (HOK1 and HOK2) and tonsil epithelia (HTEpiC) were obtained from ScienCell Research Laboratories. The LSCC cell lines were maintained in RPMI 1640 medium supplemented with 10% FBS, 200 units/ml penicillin G sodium, 200 μg/ml streptomycin sulfate and 0.5 μg/ml amphotericin B. HOK1 and HOK2 were maintained in oral keratinocyte medium (ScienCell Research Laboratories) together with oral keratinocyte growth supplement (ScienCell Research Laboratories). HTEpiC was maintained in tonsil epithelial cell medium (ScienCell Research Laboratories) supplemented with tonsil epithelial growth supplement (ScienCell Research Laboratories). Everolimus were obtained from Sigma-Aldrich.

### Tissue samples

Forty-one paired LSCC and the corresponding normal epithelia were obtained from the First Affiliated Hospital, Sun Yat-sen University, Guangdong, China. Six paired LSCC tissues were obtained from Department of Surgery, Queen Mary Hospital, The University of Hong Kong. Consent of tissue donation for research purpose was obtained before tissue collection. Tissues were collected from patients with no prior treatment including radiotherapy and/ or chemotherapy. All the tissues were snap-frozen in liquid nitrogen and were stored at −80°C until use. The protocol was approved by the human research ethics committee of Sun Yet-sen University (reference number: 2013_23) and University of Hong Kong (reference number: UW 10-142).

### MicroRNA microarray

**Figure 8:**
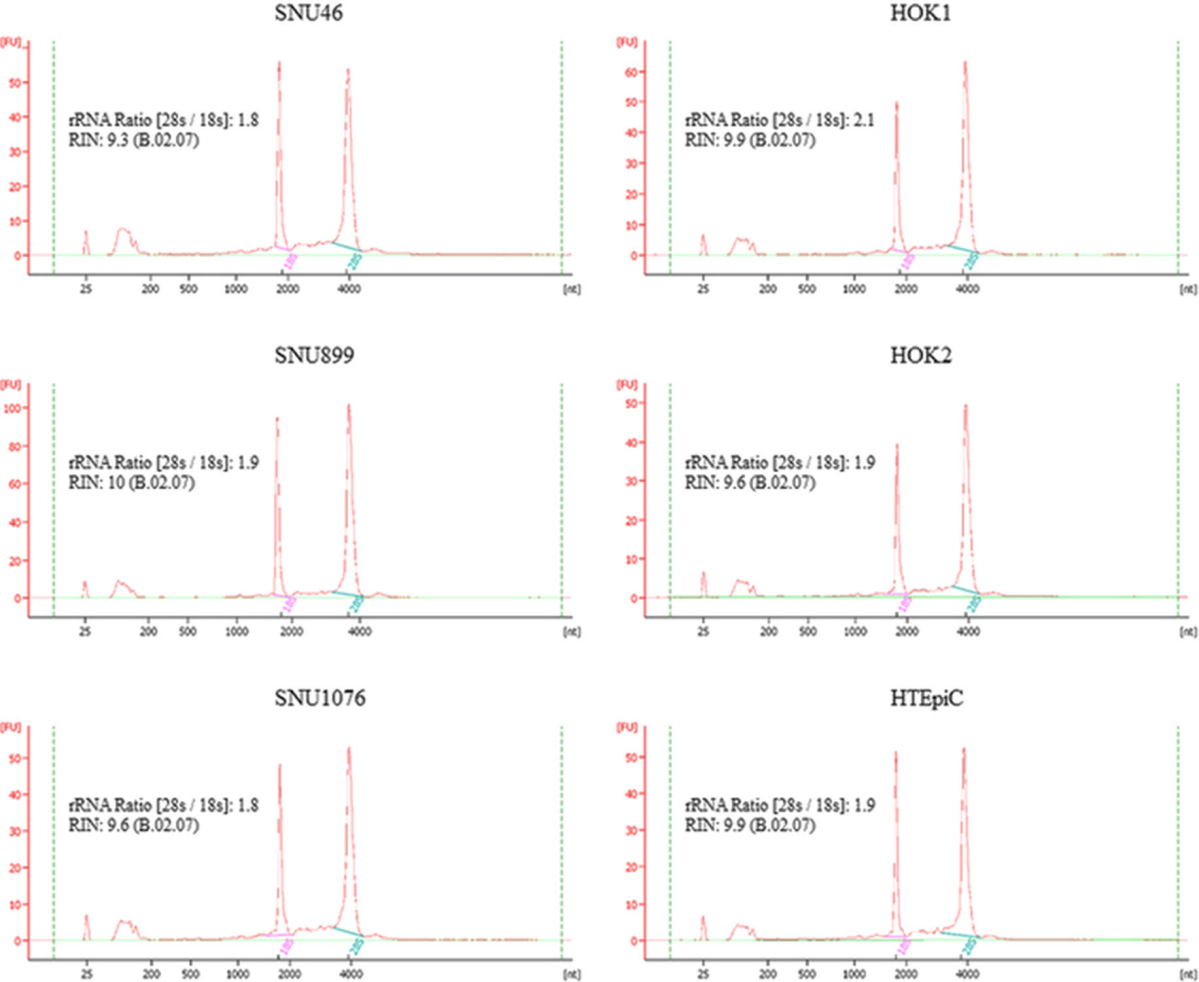
The RNA quality of the cell lines for microRNA microarray profiling All the samples had rRNA Ratio (28s/18s) over 1.8 and RNA Integrity Number (RIN) over 8.0.

RNA quality was evaluated using Agilent 2100 bioanalyser (Agilent Technologies) prior to microarray experiment. All the samples had rRNA Ratio (28s/18s) over 1.8 and RNA Integrity Number (RIN) over 8.0 (Figure 8). The experiments were performed in the Centre for Genomic Sciences, The University of Hong Kong. Data were analyzed by miRNA QC Tool software (Affymetrix). MicroRNA with *P* < 0.05 and 1.5-fold difference were considered as deregulated microRNA in LSCC.

### Quantitative reverse transcription polymerase chain reaction (QPCR)

Total RNA from the cell lines and tissues were extracted with TRIZOL reagent (Invitrogen). For microRNA cDNA preparation, microRNA was polyadenylated by using the Poly (A) Polymerase Tailing Kit (Illumina). The cDNA product was then generated using High-Capacity cDNA reverse transcription kit (Invitrogen) following the methods described by Reichenstein et al. [51]. For conventional cDNA conversion, mRNA was reversely transcribed into cDNA using High Capacity cDNA Reverse Transcription Kit (Invitrogen). Real-time PCR were performed using FastStart Universal Probe Master (Roche Applied Science) on LightCycler^®^ 480 (Roche Applied Science). Reaction was performed at 95°C for 10 minutes followed by 45 cycles of 95°C for 15 seconds and 60°C for 1 minute. Table 5 listed all the primers and the probes used in this study.

**Table 5:** Information of probe and primer for microRNA and gene measurement

MicroRNA/Gene symbol	UPL probe	Forward primer (5′–3′)	Reverse primer (5′–3′)
U6	61	ATGACACGCAAATTCGTGAAGC	GCGAGCACAGAATTAATACGAC
hsa-miR-7-1	61	CAACAAATCACAGTCTGCCATA	GCGAGCACAGAATTAATACGAC
hsa-miR-196a	61	TAGGTAGTTTCATGTTGTTGGG	GCGAGCACAGAATTAATACGAC
hsa-miR-196b	61	TAGGTAGTTTCCTGTTGTTGGG	GCGAGCACAGAATTAATACGAC
hsa-miR-744-3p	61	CTGTTGCCACTAACCTCAACCT	GCGAGCACAGAATTAATACGAC
hsa-let-7a-3p	61	CTATACAATCTACTGTCTTTC	GCGAGCACAGAATTAATACGAC
hsa-miR-34a-3p	61	CAATCAGCAAGTATACTGCCCT	GCGAGCACAGAATTAATACGAC
hsa-miR-338	61	AACAATATCCTGGTGCTGAGTG	GCGAGCACAGAATTAATACGAC
hsa-miR-365-5p	61	AGGGACTTTCAGGGGCAGCTGT	GCGAGCACAGAATTAATACGAC
PTEN	59	GCTACCTGTTAAAGAATCATCTGGA	CTGGCAGACCACAAACTGAG
PDCD4	10	TGGAAAGCGTAAAGATAGTGTGTG	TTCTTTCAGCAGCATATCAATCTC
MMP-9	6	GAACCAATCTCACCGACAGG	GCCACCCGAGTGTAACCATA

### Generation of stable LSCC clone expressing miR-744-3p shRNA or PDCD4

A stem-loop shRNA with imperfect complementation was constructed according to the antisense miR-744-3p sequence (5′-GACAACGGTGATTGGAGTTGGA-3′). The miR-744-3p shRNA coding sequence was cloned into lentivector pGreenPuro (System Biosciences) to generate the miR-744-3p shRNA expressing vector (pGreenPuro-miR-744-shRNA vector). PDCD4 insert for construction of stable LSCC clone expressing PDCD4 was amplified using Roche FastStart High Fidelity PCR System (Roche Applied Science). The forward primer for PDCD4 insert amplification was: 5′- GCA TAATGGATGTAGAAAATGAGCA-3′, and the reverse primer was: 5′- CGTACTCAGTAGCTCTCTGGTTTAA −3′. The PDCD4 coding sequence was cloned into lentivector pCDH-CMV-MCS-EF1-copGFP (System Biosciences) generating the pCDH-PDCD4 vector. The pGreenPuro-miR-744-shRNA or pCDH-PDCD4 vector as well as their mock vectors was further packaged into lentiviral particles by 293-FT cells (Life technologies) using the Lenti Starter Kit (System Biosciences). Subsequently, the lentiviral particles were transduced into the LSCC cell lines SNU899 and SNU1076 by TransDux™ (System Biosciences).

### Real-time migration and invasion assays

xCELLigence Real-Time Cell Analyzer (Roche Applied Science) was used to measure the changes in migration and invasion ability of LSCC cells in response to the changes in miR-744-3p or PDCD4 levels. CIM-Plate 16 was adopted for the migration and invasion assay. The dynamic changes of cell number (expressed as cell migration index) from upper chamber (coated with fibronectin) to lower chamber due to the migratory capacitance of LSCC cells were measured continuously over 24 hours. For the measurement of invasion propensity, the upper chamber of CIM-Plate 16 was coated with Matrigel (1:30 dilution; BD Biosciences) for 4 hour at 37°C before cell seeding. Subsequently, the dynamic invasion of LSCC cells through the Matrigel layer was recorded and expressed as cell invasion index.

### *In silico* microRNA target gene prediction

MiRWalk (http://www.umm.uni-heidelberg.de/apps/zmf/mirwalk/) was used to predict the candidate transcripts which are targeted by miR-744-3p [52]. MiRWalk retrieves predicted target gene lists from 12 databases (DIANA-microTv4.0, DIANA-microT-CDS, miRanda-rel2010, mirBridge, miRDB4.0, miRmap, miRNAMap, PicTar2, PITA, RNA22v2, RNAhybrid2.1 and Targetscan6.2). Mature miR-744-3p sequence, MIMAT0004946 was used in the computational analysis. Genes simultaneously reported by 2 or above databases were selected. In addition, gene list was generated from literature. Genes reported to be downregulated in LSCC were selected and generate the second gene list. The common genes in the 2 gene lists were further filtered using the machine learning regression method mirSVR scoring method, which measures the likelihood of target mRNA down-regulation by the microRNA from sequence and structure features in microRNA/mRNA predicted target sites [53]. A lower mirSVR score implies a greater likelihood of the downregulation of potential target genes due to specific microRNA. Gene with a mirSVR score less than −0.01 was selected for subsequent validation.

### Western blot analysis

Western blot was performed using Mini-PROTEAN^®^ Tetra Cell Systems (Bio-Rad) on 10% SDS-PAGE. Proteins were electroblotted onto polyvinylidine difluoride (PVDF) membranes (Immobilon, Millipore) by semi-dry transfercell (Bio-Rad). Membranes were incubated with PTEN (D4.3) XP^®^ rabbit monoclonal antibody (Cell Signaling) or PDCD4 (D29C6) XP^®^ rabbit monoclonal antibody (Cell Signaling) or Phospho-Akt (Ser473) antibody (Cell Signaling) or Akt (C67E7) Rabbit monoclonal antibody (Cell Signaling) or phospho-IKKα/β (Ser176/180) (16A6) rabbit monoclonal antibody (Cell Signaling) or phospho-IκBα (Ser32/36) (5A5) mouse monoclonal antibody (Cell Signaling) or NF-κB p65 (L8F6) mouse monoclonal antibody (Cell Signaling) or IKKα/β (H-470) rabbit polyclonal antibody (Santa Cruz) or IκB-α (C-21) rabbit polyclonal antibody (Santa Cruz) at 1:1,000 dilution, or phospho-NF-κB p65 (Ser536) (93H1) rabbit monoclonal antibody (Cell Signaling) or MMP-9 (D6O3H) XP^®^ Rabbit monoclonal antibody (Cell Signaling) at 1:500 dilution, or beta-actin specific antibody (Sigma-Aldrich) at 1:10,000 dilution at 4°C overnight. Signals were developed using chemiluminescence Plus Western Blotting Detection System (Amersham) and analyzed using ImageJ software version 1.48 (http://rsbweb.nih.gov/ij/).

### Argonaute 2 (Ago2) protein immunoprecipitation assay

Interaction between miR-744-3p and its target genes was confirmed by microRNA:mRNA immunoprecipitation using anti-Ago2 monoclonal antibody-immobilized beads (Wako) and human Argonaute 2 miRNA isolation kit (Wako Pure Chemical Industries). Expression levels of miR-744-3p, PTEN mRNA and PDCD4 mRNA within the Ago2-IP complex were quantitated by Real-time PCR on LightCycler^®^ 480.

### Luciferase reporter assay

The sense and antisense strands of the 3′-UTR of PTEN containing the binding sites of hsa-miR-744- 3p were synthesized. The oligonucleotides for wild-type 3′-UTR of PTEN were as follows: sense strand: 5′-CCACATCCTACCCCTTTGCACTGGCAACAGATA AGTTTGCAGTTGGCTAA-3′; antisense strand: 5′- AGC TTTAGCCAACTGCAAACTTATCTGTTGCCAGTGCA AAGGGGTAGGATGTGGACT-3′. The two single strands of the 3′- UTR of PTEN harboring a mutation in the binding sites of hsa-miR-744-3p were also synthesized. The oligonucleotides for mutant 3′-UTR of PTEN were as follows: sense strand: 5′-CCACATCCTACCCCTTT GCACCTATCGTCGATAAGTTTGCAGTTGGCTAA-3′; antisense strand: 5′-AGCTTTAGCCAACTGCAAACTT ATCGACGATAGGTGCAAAGGGGTAGGATGTGGAG CT-3′. The sense and antisense strands of wild-type and mutant 3′-UTR of PTEN were annealed and cloned into the SacI and HindIII sites of the pMIR-REPORT Luciferase vector (Applied Biosystems) to generate Luc-wild-type vector and Luc-mutant vector respectively. The Luciferase vectors containing the binding sites for miR-744-3p in 3′-UTR of PDCD4 with or without mutation were constructed using the above methods. The oligonucleotides for wild-type 3′-UTR of PDCD4 were as follows: sense strand: 5′-CGGTGCCATTGCTC TCGTTTGGGCAACAAGAGTGAAACTCTTGTCTCA AAA-3′; antisense strand: 5′-AGCTTTTTGAGACAA GAGTTTCACTCTTGTTGCCCAAACGAGAGCAATG GCACCGAGCT-3′. The oligonucleotides for mutant 3′-UTR of PDCD4 were as follows: sense strand: 5′-CGG TGCCATTGCTCTCGTTTGTATCGAGAGAGTGAAAC TCTTGTCTCAAAA-3′; antisense strand: 5′-AGCTTTTT GAGACAAGAGTTTCACTCTCTCGATACAAACGAG AGCAATGGCACCGAGCT-3′. MiR-744-3p silencing LSCC cells and their controls were seeded in 96 well plates for 24 hour before transfection. Cells were co- transfected with 50 ng of Luc-wild-type vector or Luc-mutant vector, along with 50 ng of pMIR-REPORT β-galactosidase control vector (Applied Biosystems) using Lipofectamine™ 2000 (Invitrogen). Firefly luciferase and β-galactosidase activities were measured 48 hour after transfection by Dual-Light luminescent reporter gene assay kit (Applied Biosystems, Waltham, Massachusetts, USA). Luciferase activity was normalized against β-galactosidase activity. The assay was performed in an LB 96V microplate luminometer (EG & G Berthold).

### Animal models

LSCC cells (5 × 10^5^) were injected intravenously into the tail veins of athymic nu/nu mice (5-weeks old, weight range: 18–22 g). The animals were euthanized on day 60 after injection and the lung were removed for histological examination. Hematoxylin and Eosin (H&E) staining was performed on the lung sections to evaluate the number of metastatic foci. The protocol for animal study was approved by the Institutional Committee on the Use of Live Animals in Teaching and Research (Protocol number 3675–15).

### Statistical analysis

Statistical analyses were performed using the SPSS 18.0 software package (SPSS Inc.). All results were presented as mean ± SE from three or more independent experiments. The difference between primary tumors and their normal counterparts was calculated by Wilcoxon Signed Rank test. Chi-square test and Pearson correlation test was used for evaluation on the clinical significance of microRNAs or genes in LSCC. The difference between control groups and engineered cell lines were analyzed used student-*t*-test. All the tests were 2-sided. Unless otherwise noted, *P* value < 0.05 was considered as statistically significant.
